# Butoxy Mansonone G Inhibits STAT3 and Akt Signaling Pathways in Non-Small Cell Lung Cancers: Combined Experimental and Theoretical Investigations

**DOI:** 10.3390/cancers11040437

**Published:** 2019-03-28

**Authors:** Panupong Mahalapbutr, Piyanuch Wonganan, Warinthorn Chavasiri, Thanyada Rungrotmongkol

**Affiliations:** 1 Structural and Computational Biology Research Unit, Department of Biochemistry, Faculty of Science, Chulalongkorn University, Pathumwan, Bangkok 10330, Thailand; p.mahalapbutr@gmail.com; 2 Department of Pharmacology, Faculty of Medicine, Chulalongkorn University, Pathumwan, Bangkok 10330, Thailand; 3 Center of Excellence in Natural Products Chemistry, Department of Chemistry, Faculty of Science, Chulalongkorn University, Pathumwan, Bangkok 10330, Thailand; warinthorn.c@chula.ac.th; 4 Ph.D. Program in Bioinformatics and Computational Biology, Faculty of Science, Chulalongkorn University, Pathumwan, Bangkok 10330, Thailand

**Keywords:** non-small cell lung cancer, mansonone G, apoptosis, STAT3, Akt, molecular dynamics simulation

## Abstract

Epidermal growth factor receptor (EGFR) is the key molecular target for non-small cell lung cancer (NSCLC) due to its major contribution to complex signaling cascades modulating the survival of cancer cells. Targeting EGFR-mediated signaling pathways has been proved as a potential strategy for NSCLC treatment. In the present study, mansonone G (MG), a naturally occurring quinone-containing compound, and its semi-synthetic ether derivatives were subjected to investigate the anticancer effects on human NSCLC cell lines expressing wild-type EGFR (A549) and mutant EGFR (H1975). In vitro cytotoxicity screening results demonstrated that butoxy MG (MG3) exhibits the potent cytotoxic effect on both A549 (IC_50_ of 8.54 μM) and H1975 (IC_50_ of 4.21 μM) NSCLC cell lines with low toxicity against PCS201-010 normal fibroblast cells (IC_50_ of 21.16 μM). Western blotting and flow cytometric analyses revealed that MG3 induces a caspase-dependent apoptosis mechanism through: (i) inhibition of p-STAT3 and p-Akt without affecting upstream p-EGFR and (ii) activation of p-Erk. The 500-ns molecular dynamics simulations and the molecular mechanics combined with generalized Born surface area (MM/GBSA)-based binding free energy calculations suggested that MG3 could possibly interact with STAT3 SH2 domain and ATP-binding pocket of Akt. According to principal component analysis, the binding of MG3 toward STAT3 and Akt dramatically altered the conformation of proteins, especially the residues in the active site, stabilizing MG3 mainly through van der Waals interactions.

## 1. Introduction

Lung cancer is the first leading cause of cancer-related mortality worldwide [1,2,3]. Approximately 85% of diagnosed lung cancer cases are classified as non-small cell lung cancer (NSCLC), for which the 5-year survival rate is only 17.8% [4]. The genetic alterations of epidermal growth factor receptor (EGFR) such as overexpression, exon 19 deletion, and exon 21 L858R substitution have been characterized as oncogenic drivers for NSCLC development [5,6,7]. EGFR (also known as HER1 or ErbB1), a receptor tyrosine kinase, is commonly overexpressed in several types of cancer, including lung carcinoma [8]. The activation of EGFR-mediated signaling pathways is triggered by the binding of growth factors, including EGF and transforming growth factor alpha (TGFα), to the extracellular portion of EGFR, which subsequently induces receptor dimerization and cross-phosphorylation of specific tyrosine residues located on the cytoplasmic tyrosine kinase (TK) domain. These structural modifications result in stimulating a vast array of downstream signaling cascades, e.g., mitogen-activated protein kinase (MAPK), phosphoinositide 3-kinase (PI3K)/Akt, and signal transducer and activator of transcription (STAT) pathways, leading to cell growth, proliferation, migration, and apoptosis evasion [8,9]. 

Cisplatin (CDDP), a platinum-based chemotherapeutic drug, has been commonly used to treat a wide range of solid malignancies, including lung cancer [10,11]. CDDP is the standard regimen in the first-line chemotherapy for patients with advanced stage NSCLC [10,12,13,14], especially patients carrying wild-type EGFR [15,16]. After cellular uptake, CDDP becomes a positively charged aquo complex that can interact with deoxyribonucleic acid (DNA) to form intra- and inter-strand cross-links, resulting in apoptosis induction [10,17]. However, the efficacy of CDDP-based chemotherapy is limited by numerous severe side effects [18], as well as acquired drug resistance [19,20,21,22].

The first-generation tyrosine kinase inhibitors (TKIs, e.g., erlotinib and gefitinib) have shown to significantly prolong the progression-free survival of NSCLC patients harboring EGFR mutations, primarily exon 19 deletion and exon 21 L858R substitution mutations [23,24]. TKIs compete with adenosine triphosphate (ATP) at the ATP-binding site of the receptor, inhibiting EGFR-mediated signal transduction [25]. However, acquired resistance caused by the secondary mutation T790M develops inevitably after a median response duration of 9 to 13 months [26,27,28]. The replacement of threonine (T) to methionine (M) causes a steric hindrance inside ATP-binding pocket and alters the conformation of TK domain, resulting in increasing its affinity for ATP substrate while decreasing the binding affinity for TKIs [28,29]. Given that NSCLC cells rapidly acquire resistance to both CDDP and TKIs; thus, there is an urgent need to search for a novel compound that can potentially overcome such problems by targeting alternative intracellular survival signaling pathways in NSCLC.

**Figure 1:**
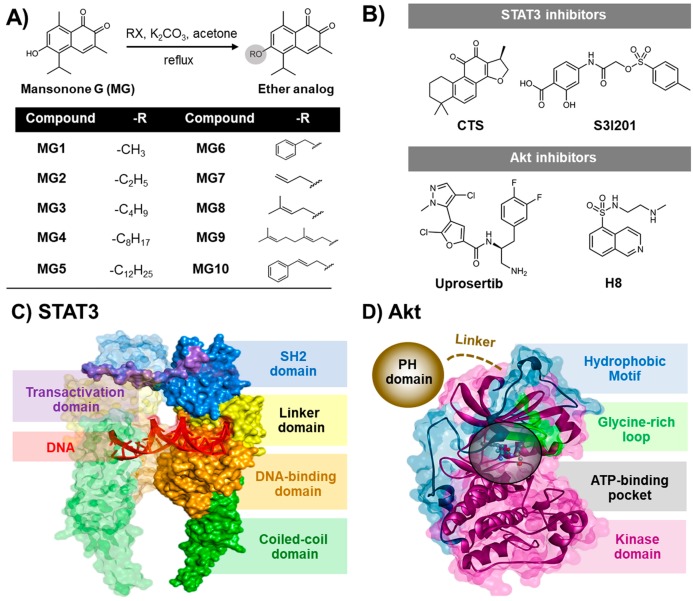
Two-dimensional (2D) chemical structures of (**A**) MG and its semi-synthetic ether derivatives MG1-MG10 [30] and (**B**) the known STAT3 (cryptotanshinone (CST) and S3I201) and Akt (uprosertib and H8) inhibitors. Three-dimensional (3D) structures of (**C**) STAT3 and (**D**) Akt1 signaling proteins. The SH2 domain of STAT3 and the ATP-binding pocket of Akt are shown by blue surface and black circle, respectively.

Mansonone G (MG, Figure 1A), a 1,2-naphthoquinone-containing compound, is the major product isolated from the heartwood extract of *Mansonia gagei* Drumm. from the Sterculiaceae family [30]. MG demonstrates various biological activities, including antitumor [31], antibacterial [32], antiestrogenic [33], anticholinesterase [34], and antifungal activities [32]. Recently, semi-synthetic ether derivatives of MG (Figure 1A) have been shown to exhibit higher antibacterial activity against *Staphylococcus aureus* [30] and inhibit adipocyte differentiation and lipid accumulation [35] more than the MG parent compound. Although several pharmacological effects of MGs have been reported, the anticancer activity of MG and its derivatives against human NSCLC remains largely unknown. Therefore, in the present study, we aimed to search for the most potent cytotoxic MG analog against human NSCLC cell lines expressing wild-type EGFR (A549) and L858R/T790M EGFR (H1975). Moreover, the mechanisms underlying cell death were experimentally and theoretically investigated.

## 2. Results

### 2.1. In Vitro Cytotoxicity Screening of MG Derivatives against NSCLC Cell Lines

Initially, we screened for the most potent cytotoxic MG analogs against NSCLC cell lines expressing wild-type EGFR (A549) and L858R/T790M EGFR (H1975) using the 3-(4,5-dimethylthiazol-2-yl)-2,5-diphenyltetrazolium bromide (MTT) assay. MG derivatives displaying a percentage of cell viability at 10 μM (%CV_10 µM_) <50 were defined as potent compounds.

As shown in Table 1, MG4 exhibited the most potent cytotoxicity against A549 cells (%CV_10 µM_ was 7.93 ± 0.43) followed by MG3 (%CV_10 µM_ was 46.90 ± 1.21), indicating that these two ether analogs were the most potent compounds toward wild-type cells. In the H1975 mutant cell line, all semi-synthesized MGs (except MG9) exhibited high cytotoxic activity with %CV_10 µM_ of <50, indicating that T790M-positve NSCLC cell line was more susceptible to MG derivatives than A549 cells. Remarkably, MG3 and MG4, exhibiting strong cytotoxicity toward A549 cells, also showed great cytotoxic effects on H1975 cells with the %CV_10 µM_ of 17.04 ± 1.42 and 23.52 ± 1.44, respectively. 

Taken together, ether analogs of MG were more cytotoxic to H1975 cells than A549 cells. The two semi-synthetic ether analogs MG3 and MG4, which exhibited high cytotoxicity toward both NSCLC cell lines, were selected for further studies.

### 2.2. Butoxy Mansonone G Exhibits a Potent Cytotoxicity against NSCLC Cells 

**Figure 2:**
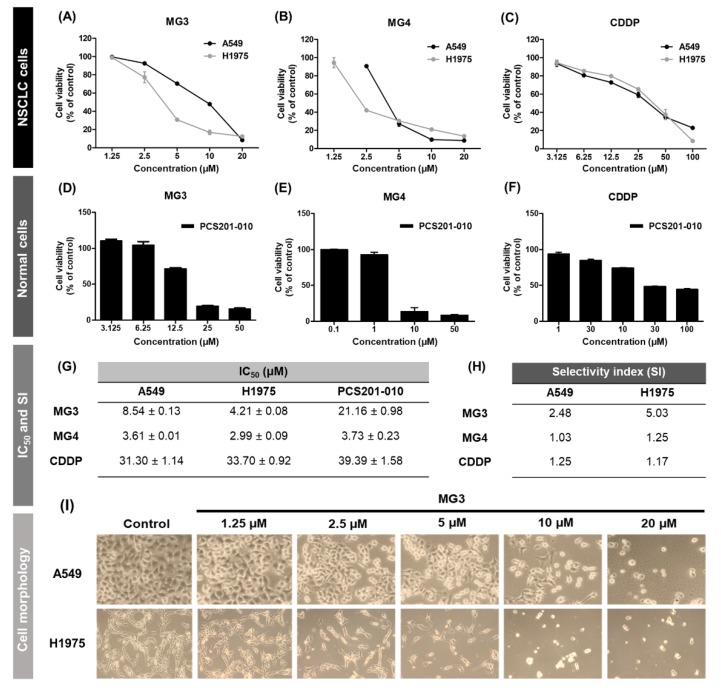
Cell viability of NSCLC (**A–C**) and PCS201-010 (**D–F**) cell lines after treatment with MG3, MG4, and CDDP for 48 h. The IC_50_ (μM) and SI of three focused compounds against all studied cell lines are shown in (**G**) and (**H**), respectively. (**I**) The morphological changes of two NSCLC cell lines treated with MG3 at various concentrations for 48 h.

The half maximal inhibitory concentration (IC_50_) of the two focused MG analogs, MG3 and MG4, and CDDP, the most commonly used chemotherapeutic agent for NSCLC patients, were evaluated using MTT assay on two NSCLC cell lines A549 and H1975. As shown in Figure 2A–C, all three tested compounds decreased the viability of both A549 and H1975 cells in a concentration-dependent manner. The IC_50_ of CDDP against two NSCLC cells obtained from this study correlated well with the previous reports [11,36]. Notably, the two selected MG derivatives were more toxic than CDDP toward cancer cells, as evidenced by the lower IC_50_ of (i) ~4-fold and ~9-fold toward A549 cells and (ii) ~8-fold and ~11-fold against H1975 cells for MG3 and MG4, respectively (Figure 2G). 

We further evaluated the toxicity of our focused MGs toward PCS201-010 normal skin fibroblast cell line (Figure 2D–F). The obtained IC_50_ results indicated that MG4 and CDDP were toxic to normal cells, as clearly shown by a similarity of IC_50_ between cancer and normal fibroblast cells (selectivity index (SI) of ~1, Figure 2H). Intriguingly, MG3 was relatively safe to normal cells, in which the IC_50_ observed in PCS202-010 cells was higher than those of A549 (SI of 2.48) and H1975 (SI of 5.03). Since MG3, possessing potent cytotoxicity against cancer cells, was less toxic to normal cells than MG4, we then elucidated the mechanisms underlying MG3-induced cell death in NSCLC cell lines experimentally and theoretically. 

According to the morphological alteration of A549 and H1975 NSCLC cells upon MG3 treatment for 48 h (Figure 2I), it can be clearly seen that MG3 dose-dependently induced cellular shrinking, a predominant characteristic of programmed cell death [37], suggesting that MG3 promoted cell death through an apoptosis-inducing effect.

### 2.3. Butoxy Mansonone G Induces Apoptosis in A549 and H1975 Cell Lines

**Figure 3:**
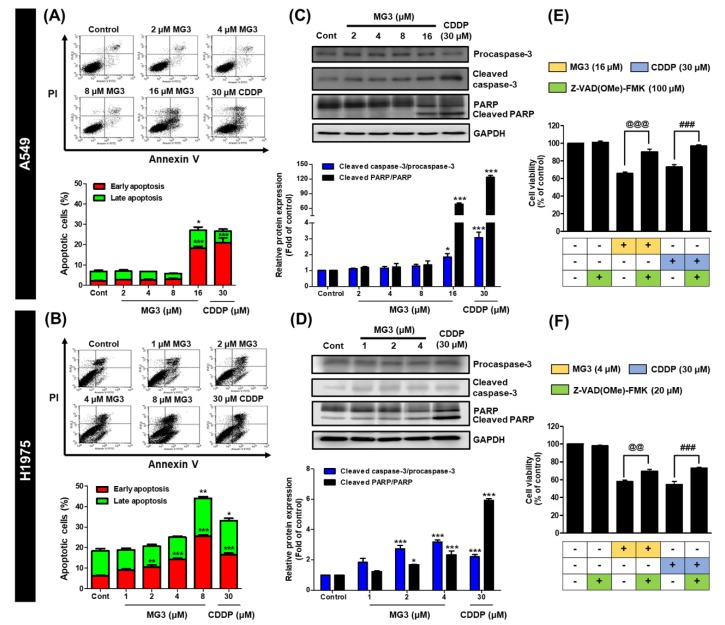
Flow cytometric analysis of Annexin V/PI stained cells after MG3 and CDDP treatments for 24 h on (**A**) A549 and (**B**) H1975 cells. Western blot analysis of apoptotic markers, caspase-3 and PARP, for (**C**) A549 and (**D**) H1975 cells. Inhibition of MG3-induced apoptosis by the pan caspase inhibitor Z-VAD(OMe)-FMK in (**E**) A549 and (**F**) H1975 cells. Data are expressed as mean ± SEM (n = 3). * *p* ≤ 0.05, ** *p* ≤ 0.01, and *** *p* ≤ 0.001 vs. control. ^@^ *p* ≤ 0.05, ^@@^ *p* ≤ 0.01, and ^@@@^ *p* ≤ 0.001 vs. MG3. ^#^ *p* ≤ 0.05, ^##^ *p* ≤ 0.01, and ^###^ *p* ≤ 0.001 vs. CDDP.

To determine whether apoptotic mechanism is involved in MG3-induced cytotoxicity in NSCLC cells, flow cytometric analysis of Annexin V/PI stained cells was carried out. Following 24 h of treatment, MG3 at 16 µM significantly induced apoptotic cell death in A549 cells, whereas MG3 at 2 µM induced significant apoptosis in H1975 cells (Figure 3A,B), indicating that apoptosis-inducing effect of MG3 was more pronounced in NSCLC cells carrying mutant EGFR than in NSCLC cells carrying wild-type EGFR. Additionally, MG3 at 8 μM dramatically induced apoptosis in H1975 cells (~50%) which was higher than CDDP, reflecting a strong cytotoxic activity. It should also be noted that, when compared to 30 μM CDDP, the lower concentrations of MG3 at: (i) 16 μM (~2-fold lower) for A549 cells and (ii) 2 μM (15-fold lower) for H1975 cells can significantly trigger cell apoptosis. 

To further confirm the apoptosis-inducing effect of MG3 on NSCLC cells, the cleavage of procaspase-3 and poly(ADP-ribose) polymerase (PARP), key hallmarks of apoptosis, was determined using western blotting. Note that for H1975 cell line, MG3 at 8 μM was highly toxic to the cells (as evidenced by flow cytometric analysis), leading to a low concentration of extracted proteins; and thus, this concentration was excluded from this study. As shown in Figure 3C,D, MG3 (16 μM for A549 and 2 μM for H1975) as well as 30 μM CDDP significantly induced the cleavage of procaspase-3 and PARP, which was in good agreement with a significant apoptotic cell death detected by flow cytometric analysis. 

We next characterized whether caspase-3 activation (Figure 3C,D) is mandatory for MG3-induced apoptosis. NSCLC cells were pretreated with Z-Val-Ala-Asp-(OMe)-fluoromethylketone (Z-VAD(OMe)-FMK), an irreversible pan-caspase inhibitor, for 1 h prior to challenge with MG3 for 24 h. As shown in Figure 3E,F, both MG3 and CDDP decreased cell viability by ~40% in both A549 and H1975 cells, and Z-VAD(OMe)-FMK alone did not affect the cell viability of cancer cells (%CV of ~100). Intriguingly, blockage of caspase activation by Z-VAD(OMe)-FMK inhibitor significantly restored cell viability in A549 and H1975 cells for both MG3- and CDDP-treated groups. These findings clearly demonstrated that activation of caspase-3 enzyme plays a crucial role in MG3-induced cell apoptosis.

### 2.4. Butoxy Mansonone G Inhibits STAT3 and Akt Signaling Pathways in NSCLC Cell Lines

**Figure 4:**
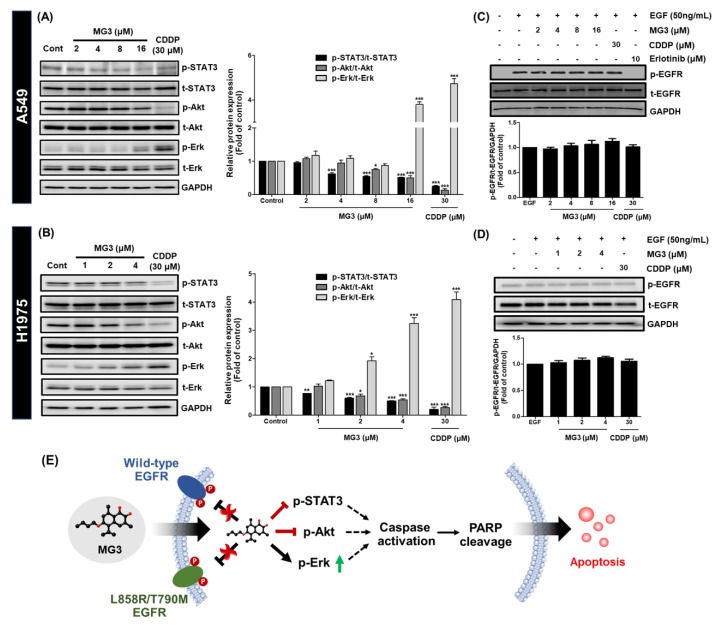
MG3 dose-dependently inhibits the phosphorylation of STAT3 (Tyr705) and Akt (Ser473) in (**A**) A549 and (**B**) H1975 cells at 24 h. The expression of p-Erk (Thr202/Tyr204) is found to be increased upon MG3 and CDDP treatments. The phosphorylation of EGFR (Y1068) for both (**C**) A549 and (**D**) H1975 cells is not significantly affected by MG3 and CDDP treatments. (**E**) Proposed mechanisms of MG3 against two studied NSCLC cell lines, in which MG3 promotes cell apoptosis through the inhibition of p-Akt and p-STAT3 as well as through the activation of MAPK signaling pathway. Data are expressed as mean ± SEM of three independent experiments. * *p* ≤ 0.05, ** *p* ≤ 0.01, and *** *p* ≤ 0.001 vs. control.

To elucidate the effect of MG3 on EGFR-mediated survival signaling pathways, western blot analysis was performed. As shown in Figure 4A,B, MG3 and CDDP significantly inhibited the phosphorylation of STAT3 and Akt in a concentration-dependent manner in both A549 and H1975 cells. Conversely, the expression of p-Erk was significantly increased after treatment with such two compounds. Remarkably, MG3, although at lower concentrations (~2-fold lower for A549 and 15-fold lower for H1975), exhibited similar effects on EGFR-mediated survival signaling pathways as 30 µM CDDP.

We further investigated whether the downregulation of p-STAT3 and p-Akt caused by MG3 was mediated through the inhibition of p-EGFR. The data in Figure 4C,D demonstrate that the addition of EGF dramatically increased p-EGFR in A549 vehicle-treated cells. On the other hand, EGF has no effect on phosphorylation of EGFR in H1975 control cells, since T790M mutation enhances the ATP binding affinity without EGF binding [38]. The 10 µM erlotinib, an EGFR TK inhibitor, totally inhibited the phosphorylation of EGFR in A549 cells, which was in agreement with a previous study [39]. However, it should be noted that pre-treatment with MG3 and CDDP did not alter the expression levels of p-EGFR following EGF stimulation, indicating that EGFR was not the preferential binding site for both MG3 and CDDP. 

Taken together, the proposed underlying mechanisms of MG3 against two NSCLC cell lines are illustrated in Figure 4E showing that MG3 inhibited downstream activity of STAT3 and Akt without interfering phosphorylation of EGFR, whilst the phosphorylation of Erk was significantly enhanced upon MG3 treatments. These signal transduction effects led to the activation of caspase-3, cleavage of PARP, and induction of apoptosis, respectively.

Since we found that MG3 inhibits p-STAT3 and p-Akt, we further elucidated the atomistic binding mechanisms of MG3 against such target proteins, which culminated in phosphorylation inhibition, using multiple computational modeling techniques. The structural and dynamics properties, ligand-protein interactions, and binding affinity of MG3 in complex with two signaling proteins were compared to the known inhibitors as well as the apo-proteins. 

### 2.5. Predictive Binding Affinity of Butoxy Mansonone G against STAT3 and Akt Signaling Proteins 

To estimate the binding affinity of MG3 against the focused proteins STAT3 and Akt in comparison with their known inhibitors, the molecular mechanics combined with generalized Born surface area (MM/GBSA) method was applied on the 200 molecular dynamics (MD) snapshots extracted from the last 200-ns simulations. The binding free energy (Δ*G*_bind_) together with its energy components are summarized in Table 2. In the case of STAT3, the calculated Δ*G*_bind_ results were ranked in the order of MG3 (−8.54 kcal/mol) << cryptotanshinone (CTS, −5.09 kcal/mol) < S3I201 (−3.73 kcal/mol), suggesting that the susceptibility of MG3 was significantly higher than those of known inhibitors. By considering Akt models, the Δ*G*_bind_ of MG3 (−9.19 kcal/mol) was in the range of uprosertib (−10.45 kcal/mol) and H8 (−9.68 kcal/mol) inhibitors. Due to the nonpolar structure of MG3 and CTS (Figure 1A,B), the molecular mechanics energy (Δ*E*_MM_) revealed that van der Waals interaction (Δ*E*_vdW_) was the main force driving protein-ligand complexation (Δ*E*_vdW_ of −35.77, −39.14, and −35.61 kcal/mol for MG3/STAT3, MG3/Akt, and CTS/STAT3, respectively). In contrast, the electrostatic attraction (Δ*E*_ele_) was found to mainly contribute toward S3I201/STAT3 (Δ*E*_ele_ of −110.36 kcal/mol), uprosertib/Akt (Δ*E*_ele_ of −146.76 kcal/mol), and H8/Akt (Δ*E*_ele_ of −168.13 kcal/mol) complexes, since these inhibitors contain the ionic charged moiety in the chemical structures (R-COO^−^, R-NH_3_^+^, and R-NH^+^-R groups, respectively, Figure 1B).

Notably, the obtained Δ*G*_bind_ of all studied inhibitors agreed well with the trend of experimental Δ*G*_bind_ (Δ*G*_bind, exp_), implying that our free energy calculations could successfully predict the binding affinity of protein/inhibitor complexes. Taken together, the experimental and theoretical results suggested that the anticancer activity of MG3 was due to binding to STAT3 and Akt signaling proteins.

### 2.6. Key Binding Residues 

**Figure 5:**
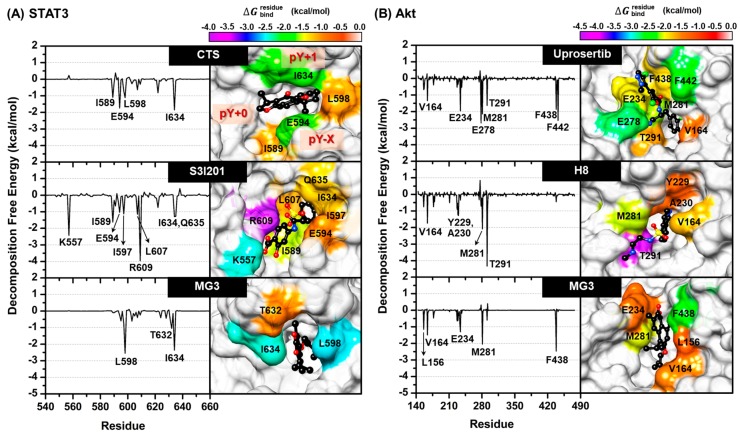
$\Delta G_{\mathrm{bind}}^{\mathrm{residue}}$ (kcal/mol) of (**A**) STAT3 and (**B**) Akt. The amino acids involved in ligand binding are shaded according to their $\Delta G_{\mathrm{bind}}^{\mathrm{residue}}$, in which the highest and lowest energies are ranged from red to magenta, respectively.

The per-residue decomposition free energy ($\Delta G_{\mathrm{bind}}^{\mathrm{residue}}$) calculation based on the MM/GBSA method was used to investigate the crucial amino acid residues involved in ligand binding within the SH2 domain of STAT3 and the ATP-binding pocket of Akt. The total contributing amino acids of all complexes are shown in Figure 5, where the negative and positive $\Delta G_{\mathrm{bind}}^{\mathrm{residue}}$ values represent respectively the stabilization and destabilization energies of the considered residue. 

In the case of STAT3, there are three subpockets in the SH2 domain, including (i) pY + 0 (residues 591 and 609–620), (ii) pY − X (residues 592–608), and (iii) pY + 1 (residues 621–639) pockets. The pY + 0 site contains several polar residues responsible for phosphotyrosine (pTyr) binding, while the two subsites pY − X and pY + 1 are the hydrophobic regions (Figure 5A). Note that among residues 458–722 of STAT3 model, only the contribution from the residues 540–660 is shown. The obtained results demonstrated that there were four and eight amino acids involved in the binding of the two STAT3 inhibitors CTS (e.g., I589, E594, L598, and I634) and S3I201 (e.g., K557, I589, E594, I597, L607, R609, I634, and Q635), respectively; whereas MG3 interacted with the residues L598, T632, and I634 inside the hydrophobic subsites pY − X and pY + 1. Notably, the contribution from residues L598 and I634 toward MG3/STAT3 complex matched to the known inhibitors. The lipophilic group of ligands, including the cyclohexane ring of CTS, the *O*-tosyl group of S3I201, and the alkyl side chain of MG3 were found to be encapsulated into the hydrophobic pY + 1/pY − X pocket of STAT3, while the polar moieties of S3I201 orientated in the pY + 0 site, forming hydrogen bond (H-bond) interactions (Figure S1).

For Akt signaling protein (Figure 5B), the amino acids establishing inhibitor binding were: (i) V164, E234, E278, M281, T291, F438, and F442 for uprosertib and (ii) V164, Y229, A230, M281, and T291 for H8. Notably, the key binding residues V164, E234, M281, and F438 involved in MG3 binding were identical to those of the Akt inhibitors. Interestingly, the ligand binding mode of Akt models shared a structurally-related characteristic, in which the aromatic moiety of all studied compounds approached the key residue M281.

### 2.7. In Silico Study on Conformational Change of STAT3 and Akt upon Butoxy Mansonone G Binding 

**Figure 6:**
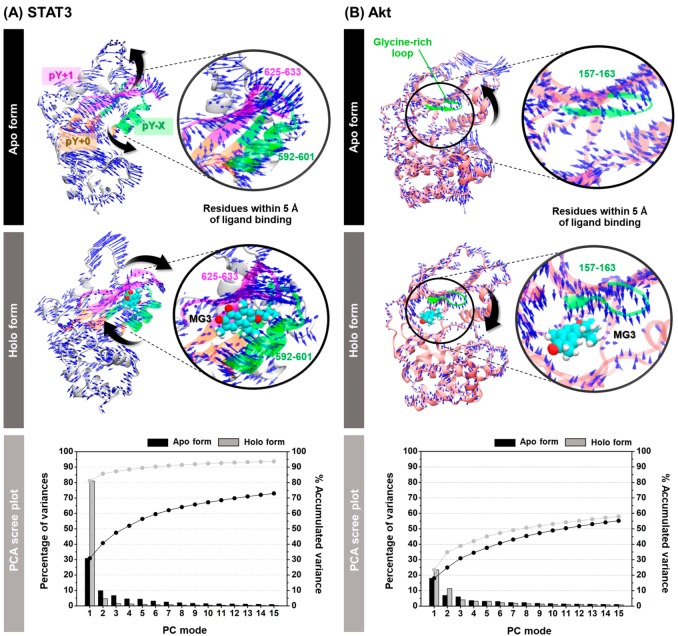
Porcupine plot of apo and holo forms of (**A**) STAT3 and (**B**) Akt across the PC1. The residues within 5 Å of ligand are presented in circle where the MG3 molecule is shown in vdW model. The PCA scree plot of quantitative characters for each protein is given below, in which the columns in black and grey represent the data for apo and holo forms, respectively.

The structurally relevant motions of STAT3 and Akt signaling proteins derived from MG3 recognition were investigated in comparison with the apo-protein using principal component analysis (PCA) on the 2000 MD snapshots taken from the last 200-ns simulations. The results are illustrated in Figure 6, where the arrow and its length indicate the direction and amplitude of motions, respectively. Note that among residues 458–722 of STAT3 model, only the protein motion from the residues 499–688 is shown. 

The first 15 PC modes showed the % accumulated variance of (i) 72.99 and 93.68 for apo and holo forms of STAT3 and (ii) 55.21 and 57.95 for apo and holo forms of Akt, respectively. The percentage of variances for PC1 of all systems was much higher than that of PC2, indicating that this mode could represent the significant motions of proteins.

By considering STAT3 model (Figure 6A), the first principal component (PC1) showed that MG3 binding importantly converted the direction of motions of overall protein to approach the ligand in a different manner from that of the apo form. Remarkably, the residues 592–601 on pY − X (green) and 625–633 on pY + 1 (magenta) hydrophobic pockets of STAT3 SH2 domain displayed not only increased direction of motions, but also enhanced amplitude of motions upon MG3 binding mainly through vdW interaction (Table 2). For the Akt systems (Figure 6B), the residues 157–163 on glycine-rich loop (GRL, green) inside the ATP-binding pocket of apo form pointed outward from the binding site, making it an opened conformation. Intriguingly, a complexation with MG3 led to the conversion of direction of GRL motion to be located closer to the MG3 molecule, resulting in a closed conformation. 

## 3. Discussion

Platinum-based chemotherapy and the first-generation TKIs have been used as the first-line treatment for NSCLC patients carrying wild-type and mutant EGFRs, respectively [15,16,23,24]. However, acquired drug resistance is inevitable after a progression-free period of approximately 9 to 13 months. Therefore, a novel anticancer compound that remains effective in both NSCLC cells expressing wild-type and T790M-positive EGFRs is critically needed. MG, a naphthoquinone-containing compound extracted from *Mansonia gagei* Drumm, was shown to exhibit anticancer activity toward the A2780 ovarian cancer cell line with an IC_50_ of 10.2 ± 0.9 μM [31]. Recently, the etherification of the hydroxyl group of MG has been reported to potentially result in antibacterial activity as well as to suppress adipocyte differentiation and lipid accumulation by more than natural MG [30,35]. In the present study, we experimentally and theoretically elucidated the cytotoxic activity of MG and its semi-synthetic derivatives against A549 (expressing wild-type EGFR) and H1975 (expressing L858R/T790M EGFR) NSCLC cell lines. We found that, among ten ether analogs, MG3 and MG4 displayed the most potent cytotoxicity toward both A549 and H1975 cells (Table 1). Notably, the IC_50_ values of such two MGs were much lower than IC_50_ of CDDP for both NSCLC cell lines (Figure 2G). Introduction of longer carbon side chain to MG makes its chemical structure more hydrophobic, which culminate in increasing cellular uptake [30,44]. Therefore, it is likely that chemical modification of MG via increasing number of carbon units of alkyl side chain enhances the cytotoxicity of MG1-MG4 against NSCLC cell lines. Conversely, MG5 containing twelve carbons showed a dramatic reduction of cytotoxic activity, which may be due to the cut-off effect [30,45]. 

Unfortunately, we found that MG4 was highly toxic toward the PCS201-010 normal skin fibroblast cell line (IC_50_ of 3.73 ± 0.23 μM), thus, this compound was excluded from our mechanistic studies. Our present findings are consistent with others showing that MG3 was less toxic to differentiated adipocytes than MG4 [35]. However, it should be noted that only one normal cell line was tested in this study; thus, further investigations on other normal cell lines as well as in vivo studies should be conducted. Interestingly, as compared to the commercial EGFR-targeted drugs, the SI of gefitinib (SI = 0.81) and osimertinib (SI = 1.15) toward A549 NSCLC and normal human bronchial epithelial (HBE) cell lines is much lower than MG3 (2.48, Figure 2H) [46]. In addition, the SI of chemotherapeutic drug methotrexate toward A549 cells and normal embryo fibroblast (NIH/3T3) cells was only 0.02, reflecting a strong toxicity [47,48]. Altogether, MG3, which showed a lower toxicity (IC_50_ of 21.16 ± 0.98 μM) against PCS201-010 cells, was then selected for further studying the mechanisms underlying cell death. 

Activation of caspase enzymes leads to the generation of signaling cascades responsible for apoptotic events [49]. In the present study, we documented that MG3 induced caspase-dependent apoptosis in both A549 and H1975 cells, in which H1975 cells expressing mutant EGFR were more susceptible to apoptosis-inducing effect of MG3 rather than A549 cells expressing wild-type EGFR. The reason for this observation is that L858R/T790M EGFR stimulates cell growth through the dramatic enhancement of catalytic phosphorylating activity over wild-type EGFR [50], which can provide more preferential target signaling proteins for MG3, as evidenced by the significant downregulation of p-STAT3 and p-Akt at low concentrations of 1 μM and 2 μM, respectively, in H1975 cells (Figure 4B). Previous studies demonstrated that CDDP induced apoptosis through the activation of caspase-3 and PARP [51,52,53,54]. Similarly, this study revealed that CDDP promoted apoptotic cell death through the activation of caspase cascades (Figure 3C–F). 

Many lines of evidence have shown that several naphthoquinone-containing compounds, such as shikonin, plumbagin, furano-1,2-naphthoquinone, ramentaceone, mansonone E, and CTS significantly inhibited STAT3 and Akt signaling pathways in various kinds of malignancies [40,55,56,57,58,59,60,61]. In agreement with these reports, data in Figure 4A,B show that our *ortho*-naphthoquinone MG3 concentration-dependently inhibited phosphorylation of STAT3 and Akt in both A549 and H1975 cell lines, which strongly correlated with MM/GBSA free energy calculations showing that MG3 could possibly interact with SH2 domain of STAT3 and ATP-binding pocket of Akt in a similar manner to that of known STAT3 and Akt inhibitors (Table 2). Remarkably, our structural analyses on both STAT3 and Akt inhibitors were consistent with other experimentally/theoretically derived data as follows. The binding orientations ((i) *O*-tosyl group of S3I201 occupying the pY − X pocket and (ii) cyclohexane group of CTS pointing toward the pY+1 hydrophobic region), key binding amino acid residues (e.g., I597, R609, and Q635 for S3I201 and I634 for CTS), and H-bond formation patterns of STAT3 inhibitors displayed a similar manner to several research works [40,62,63,64,65]. It was evidenced that M281 is the key binding residue for hydrophobic packing against indole/phenyl ring of Akt inhibitors through ε-CH···π interaction [66,67]. In correlation with this fact, our $\Delta G_{\mathrm{bind}}^{\mathrm{residue}}$ data demonstrated that the aromatic moiety of all studied ligands pointed toward M281 residue (Figure 5B). Since the α,β-unsaturated carbonyl (α,β-UC) compounds can covalently interact with biological thiols of several kinase proteins via hetero-Michael addition reaction [68], we then measured the distance between the center of mass of α,β-UC unit of MG3 and the thiol group (SH-) of cysteine residues using the final snapshot from the 500-ns MD simulation. The obtained results revealed that α,β-UC part of MG3 positioned far away (>15 Å) from the cysteine residues in Akt’s active site (Figure S2), implying that MG3 could not form the covalent adduct with Akt. Altogether, MG3 showed a somewhat similar binding pattern to STAT3 and Akt inhibitors upon molecular complexation, suggesting that MG3 could likely inhibit the phosphorylation of such proteins in a similar fashion to that of the known inhibitors. By conducting PCA, we discovered that MG3 importantly induced large conformational changes of STAT3 and Akt, especially in ligand-binding pocket, as strongly evidenced by the superimposed X-ray crystal structures between apo (PDB ID: 1GZN [69]) and holo forms (PDB ID: 4GV1 [70]) of Akt (Figure S3). This might explain the atomistic mechanisms underlying the inhibition of phosphorylation-induced activation of such signaling protein mediated by MG3, which culminate in cell apoptosis. It should be noted that in silico results from MD simulations were suggestive; thus, further experimental techniques (e.g., isothermal titration calorimetry (ITC), circular dichroism (CD), and surface plasmon resonance (SPR)) should be conducted to confirm our findings.

Although our results showed that MG3 inhibited STAT3 and Akt activities, MG3 did not interfere with the phosphorylation of EGFR, indicating that MG3 preferentially targeted EGFR’s downstream signaling molecules STAT3 and Akt rather than upstream EGFR. We also discovered that treatment with CDDP did not cause any significant changes in the expression of p-EGFR, which was in good agreement with the fact that DNA is a molecular target for CDDP [17,71], and the changes of signaling cascades are derived from platinum-DNA adduct [71]. Because PARP cleavage was more pronounced in cells treated with CDDP than cells treated with MG3, it is possible that NSCLC cells induced PARP in order to repair damaged DNA caused by CDDP-mediated platination. In contrast to CDDP, DNA was found to be the non-preferential binding site for MG3, as evidenced by: (i) the lower level of cleaved PARP than CDDP (Figure 3C,D), (ii) the higher CDOCKER interaction energy (−32.22 kcal/mol) than CDDP (−44.24 kcal/mol), and (iii) the high distance between the center of mass (C_m_) of MG3 and DNA (d(_m_(MG3)-C_m_(DNA))) obtained from three independent 100-ns MD simulations (Figure S4). Altogether, MG3 exhibited overall mechanisms of action similar to those of CDDP, but preferred targeting proteins rather than DNA, which can serve as a promising anticancer agent for NSCLC patients harboring CDDP resistance.

In contrast to STAT3 and Akt, we noticed that treatment with MG3 and CDDP induced phosphorylation of Erk in both A549 and H1975 cells. A significant upregulation of p-Erk was found to be correlated with the generation of cleaved caspase-3 and cleaved PARP (Figure 3C,D). These findings were consistent with previous studies demonstrating that high level of p-Erk can promote cell apoptosis through the activation of caspase-3 [72,73]. Erk plays a dual role in both cell proliferation and cell death [72], and activation of Erk is extremely important for CDDP-induced apoptosis [71,73,74]. Thus, it is possible that, in addition to inhibition of STAT3 and Akt, cytotoxicity of MG3 may be mediated through activation of Erk in NSCLC cells.

Altogether, our present study provided the first step of the underlying mechanisms of MG3 toward EGFR-mediated signaling pathways in NSCLC cell lines expressing wild-type and mutant EGFRs. However, further investigations on other signaling pathways as well as the kinase screening assays need to be performed in order to gain more insights into the signal transduction inhibitions caused by MG3. 

## 4. Materials and Methods

### 4.1. Experimental Part

#### 4.1.1. Chemical Reagents and Antibodies

MG was extracted from the heartwood of *M. gagei*, whereas MG ether derivatives were semi-synthesized according to the previous study [30]. Bovine serum albumin (BSA), dimethyl sulfoxide (DMSO), CDDP, MTT, and protease inhibitor were purchased from Sigma-Aldrich (St. Louis, MO, USA). RIPA lysis buffer was purchased from Thermo Fisher Scientific (Waltham, MA, USA). The protein assay reagents were purchased from Bio-Rad (Hercules, CA, USA). Human epidermal growth factor (EGF, 8916) and antibodies against phospho-EGFR (p-EGFR, 2234), total-EGFR (t-EGFR, 4267), phospho-STAT3 (p-STAT3, 9145), total-STAT3 (t-STAT3, 12640), phospho-Akt (p-Akt, 4060), total-Akt (t-Akt, 4691), phospho-Erk (p-Erk, 4377), total-Erk (t-Erk, 4695), caspase-3 (9662), PARP (9542), GAPDH (5174), and anti-rabbit IgG HRP-linked antibody (7074) were purchased from Cell Signaling Technology (Santa Cruz, CA, USA). The pan-caspase inhibitor Z-VAD(OMe)-FMK (ab120487) was purchased from Abcam (Cambridge, UK).

#### 4.1.2. Cell Lines and Culture

Human NSCLC cell lines A549 and H1975 as well as human normal skin fibroblast cell line (PCS201-010) were purchased from American Type Culture Collection (ATCC, Manassas, VA, USA). A549 cells were grown in Dulbecco’s modified Eagle’s minimal essential medium (DMEM; Gibco, Grand Island, NY, USA) supplemented with 10% fetal bovine serum (FBS; Gibco), 100 U/mL penicillin, and 100 µg/mL streptomycin (Gibco). H1975 cells were cultured in RPMI-1640 medium containing 10% FBS, 100 U/mL penicillin, and 100 µg/mL streptomycin. The DMEM with high glucose (4500 mg/L) supplemented with 10% FBS, 100 U/mL penicillin, and 100 µg/mL streptomycin was used for culturing PCS201-010 cells. All cells were maintained at 37 °C in a humidified 5% CO_2_ atmosphere.

#### 4.1.3. Cell Viability Assay

Cell viability was assessed using the MTT assay. Cells were seeded into 96-well plates at a density of 5 × 10^3^ cells/well for H1975 and PCS201-010 as well as of 3 × 10^3^ cells/well for A549. After overnight incubation, cells were treated with MGs at 10 and 100 μM for 48 h. Note that, due to the low solubility of MG4 and MG10, the highest prepared concentration was 50 μM. Subsequently, the MTT solution (5 mg/mL) was added and then incubated for 4 h. The medium was removed and 150 µL of DMSO was added to each well. Finally, the absorbance of formazan product was measured at a wavelength of 570 nm using a LabSystems Multiskan MS microplate reader (Thermo Scientific, Vantaa, Finland). The selectivity index (SI) was calculated according to the following equation: SI = IC_50_ for normal cells/IC_50_ for cancer cells.

#### 4.1.4. Western Blotting

A549 and H1975 cells were seeded into a 6-well plate at a density of 2 × 10^5^ cells/well and 3 × 10^5^ cells/well, respectively. After overnight incubation, cells were treated with indicated compounds. Note that the concentration of MG3 was varied to two-fold, one-half, and one-fourth of its IC_50_, whereas the positive control CDDP at the IC_50_ of 30 μM was used. After 24 h of incubation, cells were rinsed twice with cold PBS, homogenized in RIPA buffer containing protease inhibitor, and incubated on ice for 45 min. Total protein (20 µg) were separated on 8% SDS-PAGE and subsequently transferred to a PVDF membrane. The membrane was blocked with 3% non-fat dry milk for 1 h and then incubated with primary antibody at 4 °C overnight. After incubation, the membrane was washed thrice with TBST buffer (5 min each) and incubated with HRP-linked secondary antibody for 2 h at room temperature. Immunoreactive bands were detected using HRP substrate (Millipore, Billerica, MA, USA) and quantitatively measured using Image Studio Lite software (LI-COR, Lincoln, NE, USA). Glyceraldehyde 3-phosphate dehydrogenase (GAPDH) was used as internal control for protein normalization.

Note that for detecting p-EGFR, it was reported that the expression of p-EGFR could not be clearly detected at 24 h due to the short half-life of activated EGFR (~1.5–4 h) [75]; thus, we pre-incubated NSCLC cell lines with the indicated concentrations of MG3 and CDDP in serum free media for 1 h prior to stimulation of EGFR with EGF (50 ng/mL) for 10 min. 

#### 4.1.5. Flow Cytometric Evaluation of Apoptosis 

NSCLC cells were plated on a 6-well plate at a density of 2 × 10^5^ cells/well for A549 cells and 3 × 10^5^ cells/well for H1975 cells. After overnight incubation, cells were treated with 1, 2, 4, 8, and 16 μM MG3 and 30 μM CDDP for 24 h. After treatment, cells were harvested by trypsinization and collected by centrifugation at 1500 rpm. Subsequently, cells were washed with cold PBS and stained with 3 μL of Annexin-V fluorescein dye and 1 μL of propidium iodide (PI) at room temperature in the dark for 20 min. After that, cells were resuspended in 400 μL of cold assay buffer containing 0.01 M HEPES, 2.8 mM CaCl_2_, and 125 mM NaCl. The percentage of apoptotic cells was quantitatively measured using BD FACSCalibur flow cytometer (BD Bioscience, Heidelberg, Germany).

#### 4.1.6. Statistical Analysis

The quantitative data are expressed as mean ± standard error of mean (SEM) of triplicate experiments. Differences between groups were determined using one-way analysis of variance (ANOVA) followed by a Turkey post hoc test. Differences were considered to be significant at *p* ≤ 0.05.

### 4.2. Computational Part

#### 4.2.1. Preparation of Initial Structures 

The crystal structures of human STAT3 (PDB ID: 1BG1) [76] and Akt1 (PDB ID: 4GV1) [70] were obtained from Protein Data Bank (PDB). The missing amino acid residues were completed using SWISS-MODEL server [77]. The 3D structure of MG3 was obtained from a previous study [78], whereas the known inhibitors of STAT3 (CTS and S3I201) and Akt1 (uprosertib and H8) (Figure 1B) were built and subsequently optimized by the HF/6-31(d) level of theory using the Gaussian09 program [79]. The protein-ligand complexes were generated using CDOCKER module implemented in Accelrys Discovery Studio 2.5 (Accelrys Inc.) [80] with 100 docking runs. Note that for STAT3 SH2 domain, prior to perform docking, the protein was relaxed in aqueous solution by conducting a short MD simulation at 298.0 K for 100 ps (as detailed in the next section). The residues K591, R595, R609, E612, W623, and Q635 of STAT3 were defined as binding site with a docking sphere radius of 15 Å, whereas the co-crystalized inhibitor at ATP-binding pocket was used as the docking center for Akt. In addition, the MG3/DNA complex was also tested and simulated. Totally, there are nine simulated models in which the computational details of all system preparations are summarized in Table S1. 

The protonation states of all ionizable amino acids were characterized using PROPKA 3.0 [81] at pH 7.0. The electrostatic potential (ESP) charges of ligand were computed at the HF/6-31(d) level of theory, whereas the restrained ESP (RESP) charges and corresponding parameters of ligands were generated respectively using antechamber and parmchk modules in AMBER16 according to previous studies [82,83,84]. The AMBER ff14SB force field [85] was applied for protein, whilst the ligand was treated using the general AMBER force field (GAFF) [86,87,88]. The missing hydrogen atoms were added using the LEaP module. The added hydrogen atoms were then minimized using 1000 steps of the steepest descents (SD) and 2500 steps of conjugated gradient (CG) approaches. Subsequently, each system was solvated using TIP3P water model [89] in truncated octahedron periodic box with the minimum distance of 10 Å from the system surface. The systems were neutralized using Cl^−^ or Na^+^ counter ions. The minimization with the SD of 1000 steps and CG of 2500 steps was performed on the added water molecules and counter ions, and finally the entire system was wholly minimized using the same procedure.

#### 4.2.2. Molecular Dynamics (MD) Simulations and Binding Free Energy Calculations 

MD simulations of all studied complexes were performed under periodic boundary condition using AMBER 16 program. The short-range cutoff of 10 Å was employed for non-bonded interactions, whilst the long-range electrostatic interactions were treated using Particle Mesh Ewald (PME) summation method [90]. SHAKE algorithm [91] was applied to constrain all chemical bonds involving hydrogen. The prepared systems were heated up from 10.0 K to 298.0 K for 100 ps. Subsequently, the MD simulations with *NPT* ensemble were performed at this temperature until reaching 500 ns. From root-mean-square displacement (RMSD) analysis shown in Figure S5, the equilibrated MD trajectories in the last 200-ns simulations of all systems were extracted for further analysis. The cpptraj module was used to compute the structural and dynamics data, including RMSD, ligand-protein H-bond occupation, and protein motion via PCA. Binding free energy of all studied complexes was calculated using the MM/GBSA method [92]. Additionally, the per-residue decomposition free energy based on MM/GBSA approach was evaluated in order to identify crucial amino acids important for ligand recognition. 

## 5. Conclusions

The experimental and theoretical results obtained in this study shed light on the anticancer activity and its underlying mechanisms of butoxy MG against human NSCLC cell lines expressing wild-type EGFR and mutant EGFR, which might be useful to develop this compound as a novel anticancer agent and/or can be used as a theoretical guidance for designing and developing a new compound targeting STAT3 and Akt signaling pathways.

## Figures and Tables

**Table 1:** In vitro cytotoxicity screening of all MG analogs against A549 and H1975 NSCLC cell lines. Cells were treated with indicated compounds at 10 μM and 100 μM for 48 h, and cell viability was determined using MTT assay. Data are expressed as mean ± SEM of two independent experiments. MG derivatives exhibiting a percentage of cell viability at 10 μM (%CV_10 µM_) <50 were defined as potent compounds.

MG Analogs	A549		H1975	
	%CV_10 µM_	%CV_100 µM_	%CV_10 µM_	%CV_100 µM_
**MG**	95.31 ± 2.35	10.71 ± 0.95	77.28 ± 5.80	8.26 ± 0.79
**MG1**	70.55 ± 0.27	9.59 ± 1.48	40.46 ± 2.83	7.04 ± 0.22
**MG2**	63.57 ± 0.91	8.51 ± 0.66	28.33 ± 0.82	7.29 ± 0.57
**MG3**	46.90 ± 1.21	8.94 ± 0.67	17.04 ± 1.42	6.50 ± 0.28
**MG4**	7.93 ± 0.43	7.10 ± 0.22 ^a^	23.52 ± 1.44	7.42 ± 0.55 ^a^
**MG5**	121.54 ± 0.36	12.12 ± 1.71	44.35 ± 2.98	7.79 ± 0.12
**MG6**	79.56 ± 4.53	12.54 ± 1.33	20.64 ± 1.17	6.73 ± 0.15
**MG7**	71.84 ± 1.15	7.45 ± 0.04	27.53 ± 1.54	6.57 ± 0.25
**MG8**	66.59 ± 5.98	7.93 ± 0.06	20.24 ± 0.92	8.25 ± 0.32
**MG9**	110.92 ± 1.72	72.86 ± 3.75	89.84 ± 2.39	29.50 ± 2.80
**MG10**	51.00 ± 0.99	8.74 ± 0.30 ^a^	27.85 ± 2.35	23.19 ± 1.38 ^a^

^a^ The %CV was determined at 50 µM due to the low solubility of compound.

**Table 2:** The MM/GBSA Δ*G*_bind_ and its energy components (kcal/mol). The Δ*G*_bind, exp_ was calculated using the equation of Δ*G*_bind, exp_ = RTlnIC_50_, where R is the gas constant (1.985 × 10^−3^ kcal/mol/K), T is the experimental temperature (K), and IC_50_ is the half maximal inhibitory concentration (μM).

	STAT3			Akt		
	CTS	S3I201	MG3	Uprosertib	H8	MG3
**Δ*E*_ele_**	−6.80 ± 0.44	−110.36 ± 3.35	−1.56 ± 0.30	−146.76 ± 1.43	−168.13 ± 1.16	−9.43 ± 0.17
**Δ*E*_vdW_**	−35.61 ± 0.21	−37.47 ± 0.23	−35.77 ±0.22	−44.62 ± 0.20	−33.92 ± 0.18	−39.14 ± 0.17
**Δ*E*_MM_**	−42.41 ± 0.48	−147.83 ± 3.36	−37.33 ±0.37	−191.39 ± 1.48	−202.05 ± 1.18	−48.57 ± 0.24
**Δ*G*_solv, non-polar_**	−4.46 ± 0.02	−5.71 ± 0.02	−4.89 ± 0.02	−6.04 ± 0.02	−4.95 ± 0.01	−4.85 ± 0.01
**Δ*G*_solv, polar_**	23.99 ± 0.39	124.43 ± 3.15	15.67 ± 0.27	162.16 ± 1.22	176.02 ± 1.12	24.55 ± 0.15
**Δ*G*_solv_**	19.53 ± 0.39	118.72 ± 3.15	10.78 ± 0.27	156.11 ± 1.22	171.07 ± 1.12	19.70 ± 0.15
**Δ*E*_ele_+Δ*G*_solv, polar_**	17.19 ± 0.58	14.07 ± 4.59	14.11 ± 0.40	15.40 ± 1.87	7.89 ± 1.61	15.12 ± 0.22
**Δ*E*_vdW_+Δ*G*_solv, non-polar_**	−40.07 ± 0.21	−43.18 ± 0.23	−40.66 ±0.22	−50.66 ± 0.20	−38.87 ± 0.18	−43.99 ± 0.17
**−TΔ*S***	17.78 ± 1.69	25.38 ± 2.16	18.02 ± 1.93	24.82 ± 0.90	21.28 ± 0.66	19.67 ± 1.54
**Δ*G*_bind_**	**−5.09 ± 0.42**	**−3.73 ± 0.58**	**−8.54 ± 0.48**	**−10.45 ± 0.38**	**−9.68 ± 0.29**	**−9.19 ± 0.39**
**Δ*G*_bind, exp_**	**−7.26**	**−5.54**	**n/a**	**−9.19**	**−6.46**	**n/a**
**IC_50_ (μM)**	**4.6 [40]**	**86 [41]**	**n/a**	**0.18 [42]**	**18 [43]**	**n/a**

## Supplementary Materials

The following are available online at https://www.mdpi.com/2072-6694/11/4/437/s1, Figure S1: The percentage of H-bond occupation of the amino acid residues contributing to all ligands during the last 200-ns simulations within (A) SH2 domain of STAT3 and (B) ATP-binding pocket of Akt; Figure S2: The binding orientation of MG3 against Akt signaling protein taken from the last snapshot of 500-ns MD simulation; Figure S3: (A) The PCA result of Akt1 model. (B) the superimposed crystal structures between apo (PDB ID: 1GZN, black) and holo forms (PDB ID: 4GV1, pink) of Akt; Figure S4: The distance between the C_m_ of MG3 and DNA (d(C_m_(MG3)-C_m_(DNA))) of three independent simulations (MD1-3); Figure S5: RMSD plots of (A) STAT3 and (B) Akt models; Figure S6: Morphology of PCS201-010 cells after treatment with MG3 and CDDP for 24 h; Table S1: The computational details of all initial structures used for MD simulations.
