# Supplementary material for: Butoxy Mansonone G Inhibits STAT3 and Akt Signaling Pathways in Non-Small Cell Lung Cancers: Combined Experimental and Theoretical Investigations
Source: Cancers (Basel). 2019 Mar 28;11(4):437. doi: 10.3390/cancers11040437 (PMC6521096; doi:10.3390/cancers11040437)
Supplement: Supplementary file 1 [file cancers-11-00437-s001.pdf]

cancers

MDPI

Butoxy Mansonone G Inhibits STAT3 and Akt Signaling Pathways in Non-Small Cell Lung Cancers: Combined Experimental and Theoretical Investigations

Panupong Mahalapbutr, Piyanuch Wonganan, Warinthorn Chavasiri and Thanyada Rungrotmongkol

Supplementary Material

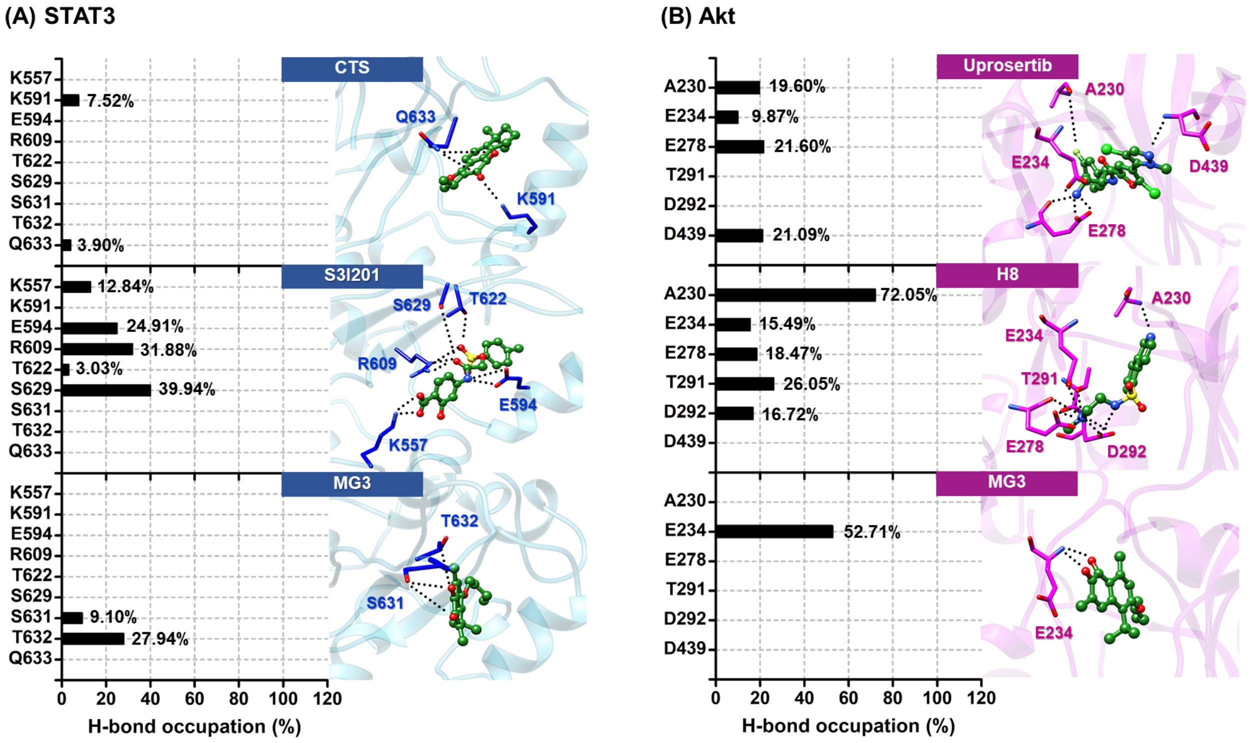

Figure S1. The percentage of H-bond occupation of the amino acid residues contributing to all ligands during the last 200-ns simulations within (**A**) SH2 domain of STAT3 and (**B**) ATP-binding pocket of Akt. Dotted lines depict the H-bond formation.

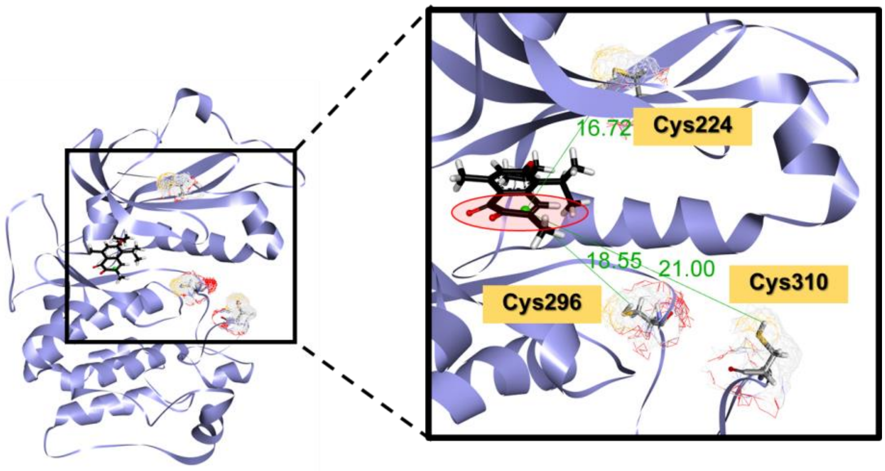

Figure S2. The binding orientation of MG3 against Akt signaling protein taken from the last snapshot of 500-ns MD simulation. The α,β-unsaturated carbonyl (α,β-UC) unit of MG3 is shown in red circle, where its center of mass (C_m) is represented in green ball. The obtained results revealed that α,β-UC part of MG3 positioned far away (>15 Å) from the cysteine residues in Akt's active site, suggesting that MG3 could not form the covalent adduct with Akt.

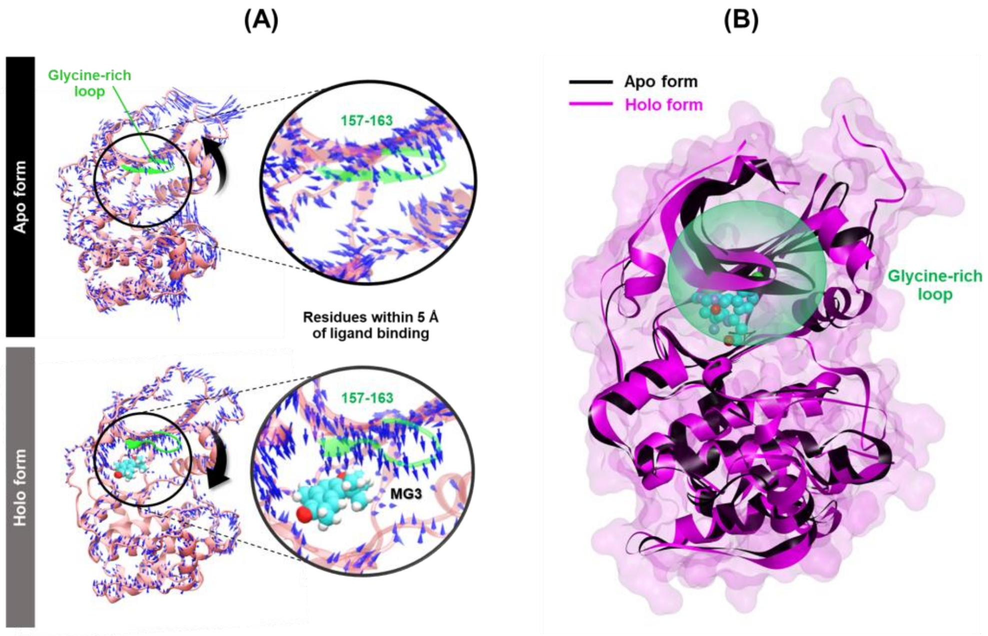

Figure S3. (**A**) The PCA result of Akt1 model. (**B**) the superimposed crystal structures between apo (PDB ID: 1GZN, black) and holo forms (PDB ID: 4GV1, pink) of Akt. Note that, due to the lack of crystal structure of Akt1 apo form in PDB data bank; thus, the apo form of Akt2 was chosen as a representative model. Our calculations agreed well with the crystal structures showing that the ligand binding induced glycine-rich loop to become significantly locate closer to stabilize ligand.

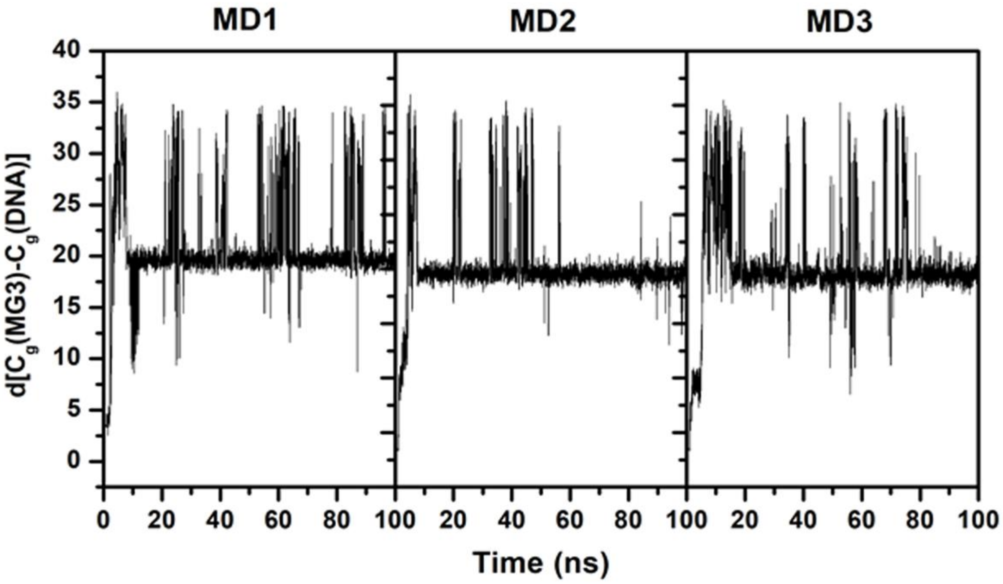

Figure S4. The distance between the C_m of MG3 and DNA ($d(C_m(MG3)-C_m(DNA))$) of three independent simulations (MD1-3).

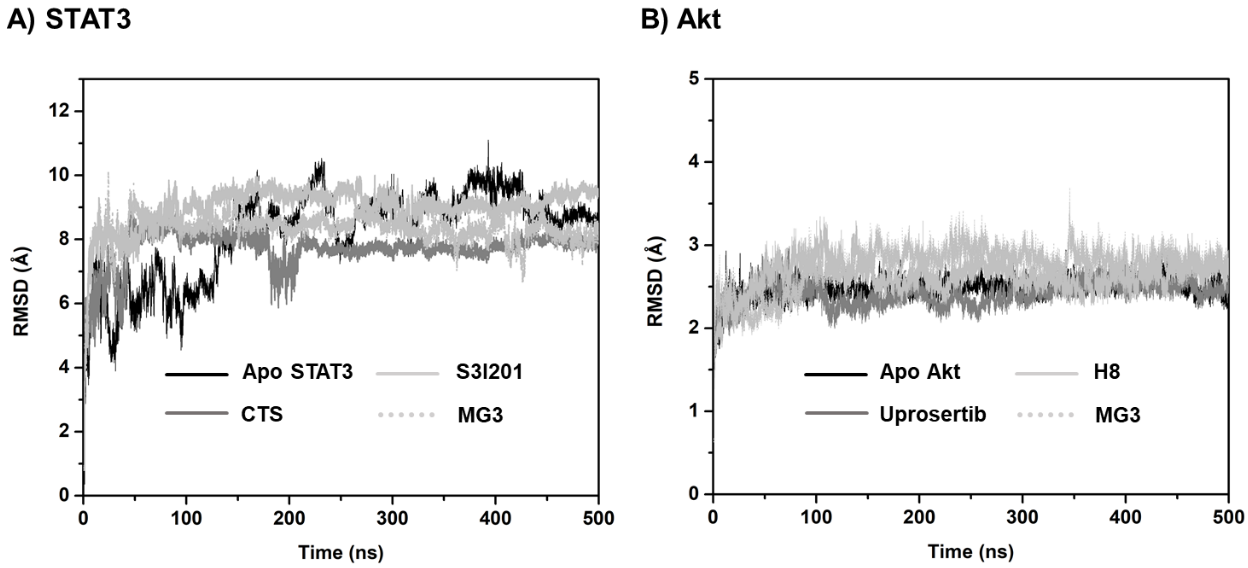

Figure S5. RMSD plots of (**A**) STAT3 and (**B**) Akt models.

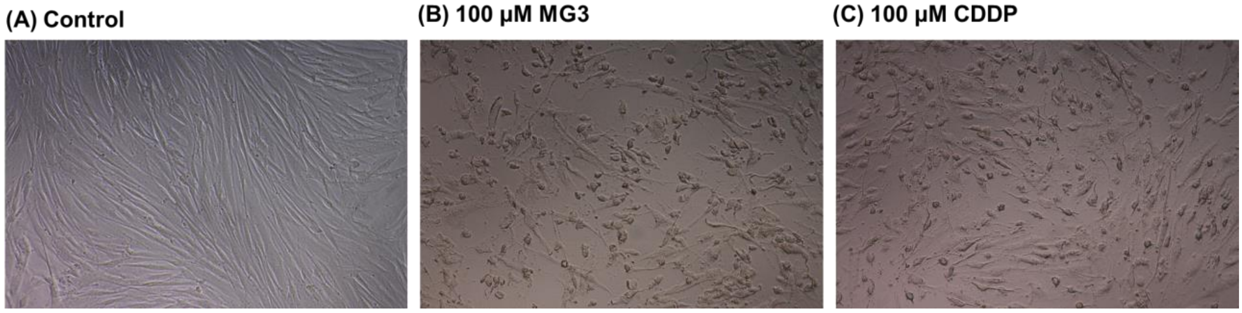

Figure S6. Morphology of PCS201-010 cells after treatment with MG3 and CDDP for 24 h. It can be clearly seen that MG3 and CDDP induced cellular shrinking, a predominant characteristic of programmed cell death, indicating that MG3 and CDDP promoted cell death through apoptosis-inducing effect.

Table S1. The computational details of all initial structures used for MD simulations.

System	PDB ID of Protein	Method for Generating Protein-Ligand Complex	Net Charge of Ligand	Amount of Added Water Molecules
**CTS/STAT3**	1BG1	CDOCKER	0	19457
**S3I201/STAT3**	1BG1	CDOCKER	−1	19339
**MG3/STAT3**	1BG1	CDOCKER	0	19398
**Uprosertib/Akt**	4GV1	CDOCKER	+1	12274
**H8/Akt**	4GV1	CDOCKER	+1	12276
**MG3/Akt**	4GV1	CDOCKER	0	12265
**STAT3 (Apo form)**	1BG1	-	-	19410
**Akt (Apo form)**	4GV1	-	-	12282
**MG3/DNA**	2NPW	CDOCKER	0	5657
